# Mechano-Signal Transduction Pathways of the Diaphragmatic Muscle and Role of Cytoskeleton

**DOI:** 10.3390/genes16080968

**Published:** 2025-08-18

**Authors:** Junaith S. Mohamed, Patricia S. Pardo, Aladin M. Boriek

**Affiliations:** 1 Laboratory of Muscle and Nerve, Department of Diagnostic and Health Sciences, College of Health Professions, University of Tennessee Health Science Center, Memphis, TN 38163, USA; 2 Center for Muscle, Metabolism and Neuropathology, Division of Rehabilitation Sciences, College of Health Professions, University of Tennessee Health Science Center, Memphis, TN 38163, USA; 3 Integrated Biomedical Sciences Graduate Program, College of Graduate Health Sciences, University of Tennessee Health Science Center, Memphis, TN 38163, USA; 4 Division of Pulmonary and Critical Care Section, Department of Medicine, Baylor College of Medicine, Houston, Texas 77030, USA; pardopatricia@gmail.com

**Keywords:** anisotropic gene regulation, mechanical stretch, mechanotransduction, skeletal muscle, cytoskeletal proteins, mechanomiRs

## Abstract

Mechanotransduction, also referred to as mechano-signal transduction, is a biophysical process wherein cells perceive and respond to mechanical stimuli by converting them into biochemical signals that initiate specific cellular responses. This mechanism is fundamental to the development and growth, and proper functioning of mechanically active tissues, such as the diaphragm—a respiratory muscle vital for breathing in mammals. In vivo, the diaphragm is subjected to transdiaphragmatic pressure, and therefore, its muscle fibers are subjected to mechanical forces not only in the direction of the muscle fibers but also in the direction transverse to the fibers. Previous research conducted in our laboratory uncovered that stretching the diaphragm in either the longitudinal or transverse direction activates distinct mechanotransduction pathways. This indicates that signaling pathways in the diaphragm muscle are regulated in an anisotropic manner. In this review paper, we discussed the underlying mechanisms that regulate the anisotropic signaling pathways in the diaphragmatic muscle, emphasizing the mechanical role of cytoskeletal proteins in this context. Furthermore, we explored the regulatory mechanisms governing mechanosensitive gene transcription, including microRNAs (mechanomiRs), within the diaphragm muscle. Finally, we examined potential links between anisotropic signaling in the diaphragm muscle and various skeletal muscle disorders.

## 1. Introduction

Mechanotransduction is a biophysical process by which cells detect their physical environment by converting physical forces and deformations into biochemical signals. These mechanosensing signaling pathways are essential for gene regulation in various cellular functions such as apoptosis, proliferation, differentiation, and migration, which are crucial for organ development and cellular homeostasis [1]. In addition, an understanding of how cytoskeleton disruption leads to dysregulation of mechanotransduction could potentially enhance our understanding of skeletal muscle diseases and disorders. Mechanically active tissues, such as muscle, always experience mechanical stress. Therefore, mechanotransduction in muscle is crucial for maintaining normal physiological functions [2,3]. This review debates the mechanotransduction pathways of diaphragm muscle, the role of cytoskeletal proteins in this process, and how abnormal mechanotransduction pathways are linked to diaphragm muscle disorders. General review articles on the mechanotransduction of tissues other than the diaphragm muscle can be found in the literature [1,3,4,5].

## 2. Diaphragm Mechanics

### 2.1. The Unique Structure of the Diaphragm Muscle

The diaphragm is a dome-shaped, thin skeletal muscle positioned at the inferior end of the thoracic cavity, separating the abdomen from the chest cavity in mammals. Mammals generally share a similar structure and functional role for the diaphragm, reflecting its specific anatomical characteristics and physiological function. The diaphragm consists of two main distinct structures: The peripheral muscular portion and the central tendon. Anatomically, the peripheral muscle fibers originate from the central tendon and insert on the lower rib cage, sternum, as well as the spine. This central, non-contractile portion is a sheet of connective tissue that provides a point of attachment for the muscle fibers [6]. The curvature of diaphragm muscle fibers plays a crucial role in converting the tension generated by the muscle into trans-diaphragmatic pressure, as well as transforming muscle shortening into diaphragmatic displacement. In mechanics, the diaphragm can be categorized as a membranous structure that can bear significant mechanical stresses in two dimensions yet is too thin to support bending moments or shear forces out of its plane. This observation indicates that alterations in diaphragm shape are limited due to the central tendon’s inherent inextensibility and the greater stiffness transverse to the muscle fibers compared to the longitudinal direction of the fibers [7]. This finding elucidates how the diaphragm muscle fibers retain consistent shapes irrespective of varying mechanical loads if such loads are within their physiologic function [7].

### 2.2. The Specific Role of the Diaphragm Muscle

The diaphragm is the primary muscle of respiration, which contracts during inspiration and or relaxes during expiration to its dome-shaped structure. Moreover, the diaphragm converts muscle tension to transdiaphragmatic pressure and muscle shortening to volume displacement. The complex geometry and kinematics of the muscle fibers of the diaphragm are crucial to its function as a respiratory pump [8]. We have shown that the diaphragm maintains its shape during physiologic loading as well as forceful respiratory maneuvers [7,9]. We have provided two mechanisms by which the diaphragm’s shape is maintained. The first is a finite element modeling approach that explains that (1) changing of diaphragm shape is restricted due to the inextensible central tendon and (2) stiffness in the direction transverse to the muscle fibers is greater than stiffness along the fibers [7]. The second is a kinematic modeling approach that showed that lateral displacement of the chest wall is a mechanism by which changes in the shape of the costal diaphragm, as described by its curvature, are limited [10].

### 2.3. In Vivo Biaxial Loading of the Diaphragm

Biaxial loading is the most obvious feature of the in vivo mechanical environment of the diaphragm, as it is continuously subjected to transdiaphragmatic pressure. Therefore, its muscle fibers experience both longitudinal and transverse mechanical loads during each respiratory cycle. It is important to recognize that any elastic sheet that is loaded uniaxially along one direction would have a different length-tension relationship than when the sheet is also loaded in the perpendicular direction. This is true for diaphragm muscles, and therefore, the mechanotransduction pathways in the diaphragm may be different from those in other skeletal muscles where the muscle fibers are oriented in one direction (longitudinal direction that is along the muscle fibers). Conducting ex vivo physiological experiments under biaxial loading of the diaphragm muscle is therefore critical in understanding the mechanotransduction of the diaphragm muscle. Our earlier in vitro biaxial loading experiments of the diaphragm have shown that the diaphragm muscle experiences a greater stiffness in the transverse direction than in the longitudinal direction to the muscle fibers [11,12]. This was consistent with our observation of negligible mechanical strains in the transverse plane of the muscle observed in vivo in the dog diaphragm. These studies suggest that there are distinct structural components in the diaphragm muscle fibers, which determine the mechanical characteristics of the diaphragm muscle in the transverse plane. Further studies in our lab have demonstrated that structural proteins play a role, in part, in mediating the anisotropic mechanical properties of the diaphragm muscle [13].

## 3. Role of Cytoskeletal Proteins in Diaphragm Mechanics

### 3.1. Cytoskeletal Proteins

**Figure 1:**
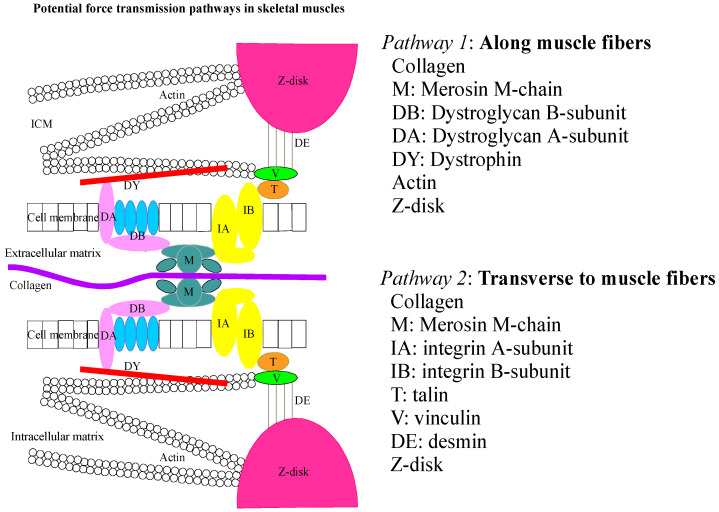
Cytoskeletal protein arrangements and their possible force transmission pathways in skeletal muscles. This schematic picture presents 2 pathways for force transmission. Pathway 1 includes the structural elements that are situated along the muscle fibers. These structural elements are collagen, merosin m-chain (M), dystroglycans B-subunit (DB), dystroglycans A-subunit (DA), sarco-glycan complex (α, β, γ, δ, ε), dystrophin (DY), actin, and z-disks. Pathway 2 consists of structural elements situated in the transverse plane to the muscle fibers. Structural elements in this pathway include collagen, merosin m-chain, integrin A-subunit (IA), integrin B-subunit (IB), talin (T), vinculin (V), desmin (DE), and z-disks. Merosin is visualized in pathway 1, along muscle fibers, and in pathway 2, transverse to muscle fibers. J Appl Physiol 94: 2524–2533, 2003 [17].

Cytoskeletal proteins are a group of proteins that form a dynamic network within cells and provide structural support, enabling cell movement and facilitating intracellular transport. Microfilaments (actin), intermediate filaments, and microtubules are the three major types of cytoskeletal proteins of eukaryotic cells. Previous research on the structure of active and passive components of muscle cytoskeletal proteins has identified two functionally and molecularly distinct categories of structural proteins. The first category comprises sarcomere proteins, which are organized into well-structured macromolecular assemblies that play a critical role in active force production by muscles (such as sarcomere actin and myosin proteins). Additionally, this category includes proteins that significantly contribute to the longitudinal passive properties of muscle, including titin and nebulin [14]. The second group contains membrane-associated structural proteins that function in part to transmit forces generated by sarcomere proteins across the cell membrane (e.g., the integrins and associated structural proteins and the dystrophin complex) [15]. The latter group is highly condensed at the ends of the muscle fibers, whereas longitudinally transmitted active and passive forces would be transmitted across the cell membrane. However, these proteins are also enriched in periodic structures, called costameres, at the lateral surface of muscle fibers, which suggests that they function in the transmission or dissipation of forces applied in the transverse plane of the muscle fibers. Although the existence of costameres implies that mechanical loading of muscle cells in the transverse plane may be a significant feature of diaphragm muscle micromechanics. This is because the diaphragm muscle fibers experience mechanical loading not only in the fiber direction but also in the transverse direction to the fibers [11,12,16]. Thus, the cytoskeletal proteins could potentially play an important role in force transmission during the in vivo biaxial loading of the diaphragm muscle. The general schematic overview of the arrangement of the various key cytoskeletal proteins and their possible roles in the direction of the force transmission pathways in skeletal muscle is shown in Figure 1 [17].

### 3.2. Desmin’s Role in the Mechanotransduction Pathways of the Diaphragm Muscle

Desmin is an intermediate filament and cytoskeletal structural protein predominantly found in the transverse plane of muscle fibers and appears to connect adjacent Z disks in parallel [18]. However, desmin intermediate filaments differ from other prominent cytoskeletal structures in muscle because they are oriented in both the transverse and longitudinal planes of the cell [18,19]. This dual orientation of desmin suggests the possibility that desmin may not only contribute to the mechanical properties in both the transverse and longitudinal planes, but desmin may also integrate the transverse and longitudinal mechanical systems [13]. Hence, desmin may integrate the three-dimensional active and passive mechanical properties of the muscle; they may contribute to mechanical properties in muscle that experiences exclusively uniaxial loading along the length of the muscle fibers and in the diaphragm muscle that undergoes biaxial loading. We showed that passive transverse stress alters the production of both maximal and submaximal contractile properties in the normal diaphragm, and this effect is absent in the diaphragm muscle that lacks desmin protein. In addition, stretching the diaphragm in the transverse plane led to a significantly more extensible muscle in the desmin-null mouse compared to the control mouse that has desmin in the muscle [13]. Furthermore, a significant reduction in the viscoelasticity of desmin null muscles compared to similar muscles from the control mouse was observed. Our results have shown significant reductions in coupling between the longitudinal and transverse properties, indicating for the first time a role for a specific protein in integrating the three-dimensional mechanical properties of muscle. Our findings offer new insight into force transmission among cells, providing an understanding of the cytoskeleton’s function within the structural and mechanical complexity of muscles [13].

### 3.3. Role of α-Sarcoglycan in Modulating the Mechanical Properties of the Diaphragm Muscle

α-sarcoglycan (ASG) is a protein that plays a crucial role in maintaining the structural integrity of muscle fibers, particularly in the sarcolemma (muscle cell membrane). It is a component of the dystrophin-glycoprotein complex (DGC), which links the muscle’s internal scaffolding (cytoskeleton) to the extracellular matrix. ASG is a transmembrane protein situated along the length of the muscle fibers. ASG is one of at least five glycoproteins that are essential to the function of the sarcoglycan complex, and its deficiency results in the downregulation of other glycoproteins. Mutations in the ASG gene cause limb-girdle muscular dystrophy type 2D, an autosomal recessive disorder [20]. Molecular analysis of the ASG-deficient mice demonstrated that absence of ASG resulted in (1) less pronounced sarcoglycan complex, (2) sarcospan (a disruption of the α-dystroglycan association with membrane recessive disorder, (3) progressive muscular dystrophy, and (4) muscle necrosis with age, a characteristic of the human muscle diseases [21,22]. ASG is a structural protein that could be a load-bearing element in the plane of the sarcolemma. ASG could be like the extracellular protein, merosin, in that it can transmit forces, mostly in shear, between the cytoskeleton and the collagen matrix fibers. Therefore, force transmission pathways in both the longitudinal and transverse direction of the myofibers may be disrupted in an ASG knockout (ASG^-/-^) mouse diaphragm. 

To determine if the absence of ASG in muscle would disturb the diaphragm’s mechanical properties, we compared the length-tension relationships of the diaphragm between the ASG^-/-^ and control mice in response to biaxial mechanical loading. We found that the diaphragm muscles of ASG^-/-^ mice displayed a significant decrease in (1) muscle extensibility, (2) contractile force-generating capacity, and (3) coupling between longitudinal and transverse properties following biaxial loading [23]. These findings provided experimental evidence that the sarcoglycan complex proteins contribute to diaphragm muscle stiffness and modulate its contractile properties. 

### 3.4. Dystrophin and Its Role in the Mechanotransduction of the Diaphragm Muscle

Dystrophin is another intracellular element of the transmembrane protein network known as DGC, which maintains the mechanical stability of the muscle fiber membrane during muscle contraction and relaxation [24,25,26]. Dystrophin is localized in the sub-sarcolemmal region of skeletal and cardiac muscle [27]. Dystrophin binds to the cytoskeletal actin and the cytoplasmic tail of transmembrane DGC protein β-dystroglycan and thus forms a part of the link from the cytoskeleton to the extracellular matrix [28,29], thereby contributing to the mechanical properties of skeletal muscle fiber. Functional inactivation of the dystrophin gene is the primary cause of Duchenne muscular dystrophy (DMD) in humans and mdx mice, a mouse model of DMD [30,31]. When compared to the length–tension relationships of the control mouse diaphragm, we found an increased muscle compliance in the dystrophin-deficient diaphragm muscle, both along and transverse to the fiber direction [32]. More recently, we compared the mechanics of dystrophin-deficient skeletal muscles between young and aged mice [33]. Our data revealed that the passive mechanics of the diaphragm muscle of the mdx mouse were significantly affected by aging. In the prefibrotic/prenecrotic stage, loss of dystrophin altered muscle extensibility and compliance, as well as viscoelasticity and muscle contractility. Consistent with these findings, data by Pasternak et al. at the single-cell level suggested an increased compliance in dystrophin-deficient myotubes, mature muscle cells in vitro [34]. These studies reveal that dystrophin deficiency leads to loss of muscle stiffness and increased extensibility.

### 3.5. Role of Integrins in the Mechanotransduction of the Diaphragm Muscle

α7β1 integrin is a transmembrane structural protein that is highly concentrated in myotendinous junctions and costameres of skeletal muscles [35,36,37]. α7-Integrin is also found along the entire sarcolemma and at myomyonal junctions in series-fibered muscles [38]. Therefore, α7-integrin may be involved in force transmission not only between the ends of muscle cells and the tendon but also between the axial and lateral elements of the muscle. It is well recognized that α7-integrin is a receptor for the laminin family of basement membrane proteins found in the extracellular matrix [39,40]. Absence of the α7-integrin subunit leads to degenerative disorders of skeletal muscles and disrupts a potential force transmission pathway through the integrin complex [41,42]. In addition, a novel form of congenital muscular dystrophy with normal expression of merosin and deficient levels of α7-integrin has been documented in humans [42]. A previous study showed that the absence of α7-integrin in mice does not alter expression of the associated dystroglycan proteins and the extracellular protein merosin, both of which provide links to the sarcolemmal membrane [43,44]. The effect of transverse passive mechanical stress on contractile force production in the diaphragm muscles of normal mice and those of mice lacking the key transmembrane protein integrin could be important to the understanding of the mechanisms of force transmission. So, the absence of α7-integrin might alter diaphragm muscle compliance and viscoelasticity as well as disrupt contractile force transmission between the longitudinal and transverse axes of the diaphragm muscle [45]. We showed that compared control mice, the diaphragm muscle of α7-integrin^-/-^ mice showed a significant (1) decrease in muscle extensibility in 1-year-old mice, whereas a robust increase in muscle extensibility in the 1-month-old mice; (2) altered muscle viscoelasticity in the transverse direction of the muscle fibers of 1-month-old mice; (3) increase in force-generating capacity in the diaphragms of 1-month-old mice, whereas in 5-month-old mice muscle contractility was depressed; and (4) reduction in mechanical coupling between longitudinal and transverse properties of the muscle fibers of 1-month-old mice [45]. These findings suggest that α7-integrin serves a vital mechanical function in the diaphragm by contributing to passive compliance, viscoelasticity, and modulation of its muscle contractile properties [45].

### 3.6. Titin’s Role in Diaphragm Muscle Mechanotransduction

Titin is a giant myofilament [46] and is critical for myofibrillogenesis [47,48] and sarcomeric integrity during contraction [49]; it confers passive elastic properties to striated muscle [50]. It is also implicated as a structural scaffolding element [51] that serves important mechanosensitive signaling functions [52]. Muscular dystrophy with myositis (mdm) is an early-onset, autosomal recessive mouse disease and was reported around the same time as the discovery of the genetic defect in DMD [53]. The titin mdm (Ttnmdm) mutation leads to aberrant splicing of four critical skeletal muscle-specific exons encoding 83 amino acids in the N2A region of the I band. The physiological importance of the titin N2A region is further underscored by the severe disease it causes in the mdm mouse model. The mdm mouse model is a pertinent clinical phenotype because it exhibits a clinical course similar to that of DMD, in that mdm mice have an early-onset, rapidly progressive muscle-wasting disease that ends in premature death, likely due to respiratory insufficiency. Hence, the titin N2A deletion in the mdm mouse diaphragm would have a deleterious impact on the force-generating capacity and altered passive mechanical properties, independent of major histopathology [54]. Our study using the costal diaphragm of the mdm mouse showed that maximum tetanic stress generation measured in response to increasing stimulation frequencies demonstrated significant weakness in two-week-old mdm diaphragms. The images of the thorax of mdm mice scanned by high-resolution micro-computed tomography (CT) confirmed the above findings [54]. Our data strongly support early contractile and passive mechanical aberrations of the respiratory pump in mdm mice. Overall, our findings from various cytoskeletal deficient mouse models provided valuable information on the role of cytoskeletal proteins in the mechanical properties, loading environment, morphometric features, and physiological function of the diaphragm muscle.

## 4. Anisotropic Mechanotransduction Pathways of the Diaphragmatic Muscle

### 4.1. Anisotropic Regulation of Signaling Pathways in Diaphragm Muscle

**Figure 2:**
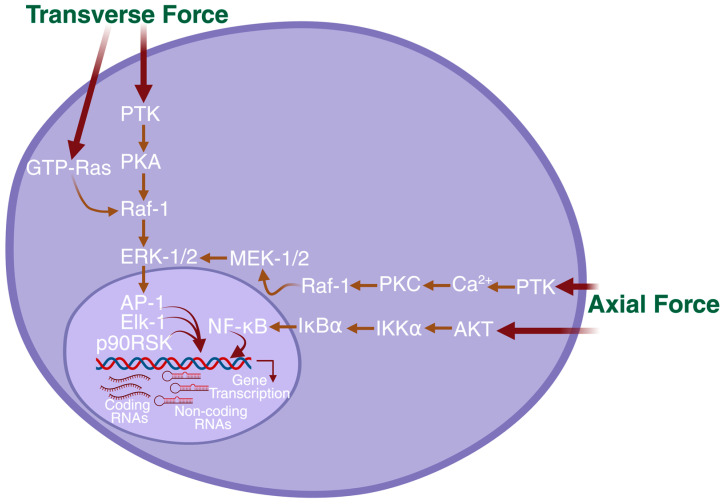
The proposed model of the anisotropic regulation of gene transcriptions in the diaphragmatic muscle. In this model, when the diaphragm is mechanically loaded in the transverse direction, the force activates AP-1, Elk-1, or p90RSK signaling proteins. In contrast, when the diaphragm is mechanically loaded in the axial (longitudinal) direction, the force activates NF-κB transcription factors through Akt or PTK signaling pathways. Subsequently, these distinct directional dependents signaling pathways trigger gene transcriptions.

It is important to determine how mechanosensitive signaling pathways are activated in the diaphragm muscle fibers in response to mechanical stretch, not only in the fiber direction but also in response to stretch applied in the direction transverse to the fibers. To do this, we conducted experiments such that mechanical stress was applied in either the direction of the muscle fibers or transverse to the fibers [55]. In our experimental protocols, axial stress applied to the muscle was essentially the same as that applied transversely; this resulted in mechanical stretch that is about twice as much in the axial direction as in the transverse fiber direction [56]. Fascinatingly, our study discovered that two distinct directionally dependent mechano-signaling pathways were activated in response to mechanical stress applied axially and transversely to muscle fibers of the diaphragm [55]. Our data showed that phosphatidylinositol 3-kinase (PI3K), protein kinase C (PKC), and mitogen-activated protein kinase 1/2 (MEK-1/2) were activated when the mechanical stress was applied axially, but were not activated when the mechanical stress was applied in the transverse direction to the muscle fibers. On the other hand, cyclic AMP-dependent protein kinase A (PKA) is activated only in response to transverse mechanical stress (Figure 2). Moreover, the greater activation of extracellular signal-regulated kinases 1 and 2 (ERK1/2), p38, rapidly accelerated fibrosarcoma-1 (Raf-1) kinases, an Ets family of transcription factors (Elk-1) and the transcription factors, activator protein-1 (AP-1) were observed in response to transverse mechanical stress, perhaps as a result of greater transverse muscle stiffness compared with axial stiffness. Other studies have shown the activation of ERKs, c-Jun N-terminal kinases (JNKs), and p38 mitogen-activated protein (MAP) kinases in skeletal muscle by mechanical stress [57,58,59,60]. We additionally showed activation of 90 kDa ribosomal S6 kinase (p90 RSK) and Elk-1, the downstream targets of ERK1/2 [55]. These observations indicate that mechanical loading of the diaphragm muscle during each respiratory cycle activates multiple intracellular signaling pathways. Most of these pathways are probably triggered by either longitudinal or transverse directional loading. Nonetheless, it remains possible that some signaling pathways are likely activated independently of stretch direction or direction of stretches. Additional research is necessary to delineate the direction-dependent and direction-independent signaling pathways in the diaphragm muscle.

### 4.2. Anisotropic Regulation of Gene Expression in Diaphragm Muscle

If distinct directional mechanical stretches activate different signaling pathways, then they may also activate either different gene transcription or activate the same gene with distinct direction-dependent mechanosensing signaling pathways. In line with the above statement, we identified that stretching in the longitudinal or transverse directions to the diaphragm muscle fibers increased the expression of ankyrin repeat domain 2 (*Ankrd2*) gene through two distinct signaling pathways in the mouse diaphragm [56]. More precisely, we found that longitudinal stretch activated Akt, also known as protein kinase B (PKB), which up-regulated *Ankrd2* gene expression through the transcription factor nuclear factor-κB (NF-κB). In contrast, transverse stretches activated rat sarcoma-guanine triphosphate (Ras-GTP), Raf-1, and ERK1/2 proteins, which up-regulated *Ankrd2* expression through AP-1 transcription factor [56]. We also found that although both directional stretches activated Raf-1, ERK1/2, and AP-1, transverse stretch activated these proteins in a higher magnitude, and this is correlated with greater transverse muscle stiffness compared with longitudinal stiffness. Furthermore, the small Rho GTPase protein GTP-Ras, an immediate upstream target of Raf-1, was activated only by transverse stretch rather than longitudinal stretch, which suggests specificity in the mechanosensing ability of this signaling protein (Figure. 2). These results suggest that activation of signaling proteins by mechanical signal transduction in the diaphragm muscle is dependent on the direction of mechanical force that applied to the diaphragm muscle fibers. Our finding suggests the anisotropic regulation of the *Ankrd2* gene expression in the diaphragm muscles via two distinct mechanosensitive signaling pathways. 

### 4.3. Anisotropic Regulation of mechanomiRs in Diaphragm Muscle

**Figure 3:**
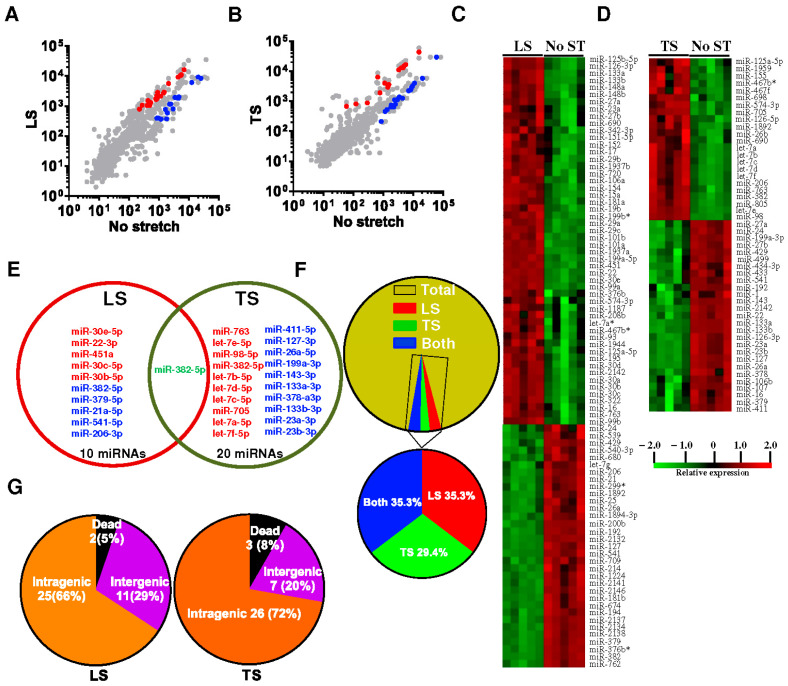
Genome-wide expression profile of mechanomiRs in diaphragm muscle by anisotropic regulation. Longitudinal or transverse stretch was applied to mouse left hemidiaphragm for 15 minutes. The right hemidiaphragm was treated as a control with no stretch. Immediately after stretch, total RNA was isolated from stretched and non-stretched diaphragm and used in miRNA microarray analyses to determine differentially regulated mechanomiRs. (**A**,**B**) The scatter plot shows log_10_-transformed signal intensities for each probe labeled with Cy3 for unstretched (control) and Cy5 for LS (**A**) or TS (**B**) diaphragm. Each dot represents one miRNA probe. (**C**,**D**) Data on the heat map show differentially expressed mechanomiRs in the diaphragm in response to LS (**C**) or TS (**D**). (**E**) Venn diagram shows up (red)- and down (blue)-regulated mechanomiRs (1.5-fold) after corresponding stretch. (**F**) Percentage of differentially expressed mechanomiRs to the total number of miRNAs in the array. (**G**) Percentage of differentially expressed mechanomiRs based on their genomic location. Asterisk (*) denotes the less abundant miRNA strand. Mohamed et al., 2015 J. Biol. Chem. 290 (41) 24986–25011 [61].

**Figure 4:**
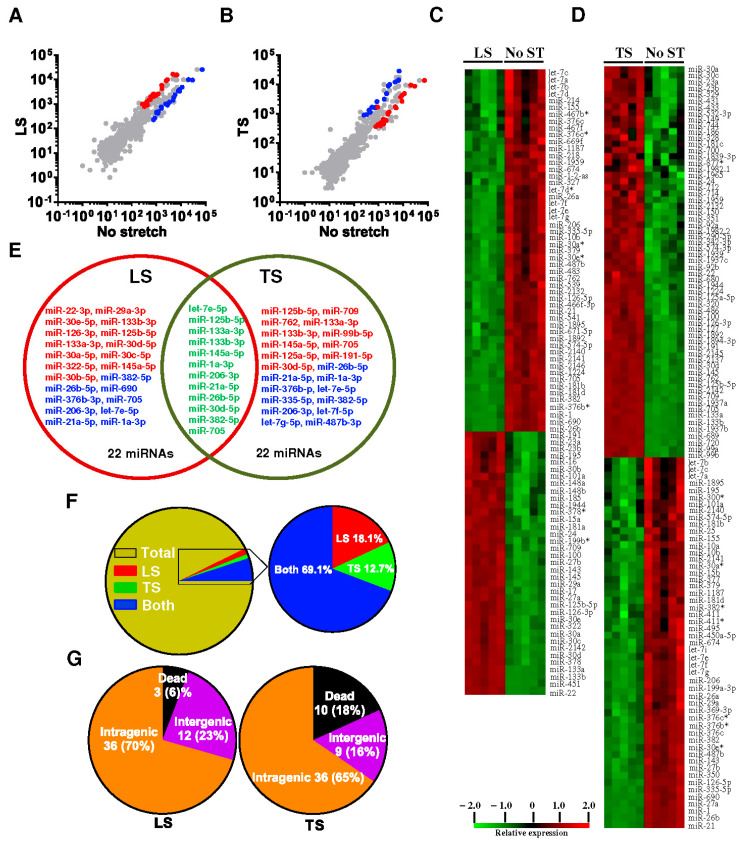
Anisotropic regulation of mechanomiRs in the diaphragm muscle of mdm dystrophic mice. Longitudinal or transverse stretch was applied to the dystrophic mouse’s left hemidiaphragm for 15 minutes. The unstretched right hemidiaphragm was treated as a control. Immediately after stretch, total RNA was isolated from stretched and non-stretched diaphragm and used in miRNA microarray analyses to determine differentially regulated mechanomiRs. (**A**,**B**) The scatter plot shows log_10_-transformed signal intensities for each probe labeled with Cy3 for unstretched (control) and Cy5 for LS (**A**) or TS (**B**) diaphragm. Each *dot* represents one miRNA probe. (**C**,**D**) Data on the heat map show mechanomiRs differentially expressed in the diaphragm in response to LS (**C**) or TS (**D**). (**E**) Venn diagram shows up (red)- and down (blue)-regulated mechanomiRs (>1.5-fold) after stretch. (**F**) Percentage of differentially expressed mechanomiRs to the total number of miRNAs in the array. (**G**) Percentage of differentially expressed mechanomiRs based on their genomic location. Asterisk (*) denotes the less abundant miRNA strand. Mohamed et al., 2015 J. Biol. Chem. 290 (41) 24986–25011 [61].

MicroRNAs (miRNAs) are small, non-coding RNA molecules that play a crucial role in the regulation of gene expression by binding to complementary sequences on target mRNAs, leading to their degradation or translational repression [61]. These short RNA sequences, typically about 22 nucleotides long, are involved in various physiological processes such as development, differentiation, apoptosis, and stress response. In the context of muscle tissues, especially the diaphragm, miRNAs play a pivotal role in modulating the response to mechanical stress and ensuring proper muscle function. The regulation of miRNAs in the diaphragm is influenced by the direction and magnitude of mechanical loading, highlighting their role in the mechanotransduction pathways that maintain muscle integrity and performance. We have recently shown genome-wide regulation of mechanosensitive microRNAs (mechanomiRs) in the diaphragm muscle, and this regulation is dysregulated in dystrophic muscle. We identified new sets of mechanomiRs from control and mdm mice diaphragms; the regulation of some of these mechanomiRs is dependent on the direction of stretch [61]. We also identified a few mechanomiRs that are independent of the direction of applied stretch. Compared with longitudinal stretch, transverse stretch induced a higher number of mechanomiRs that are entirely different from the mechanomiRs induced by longitudinal stretch (Figure 3). Although the diaphragms from control and mdm mice differentially regulated a similar number of mechanomiRs, the mdm mouse diaphragms showed a higher number of differentially regulated mechanomiRs (Figure 4).

The mechanosensing ability of the wild-type (WT) mouse diaphragm was greater in the transverse direction, possibly due to greater transverse muscle stiffness, as discussed earlier, compared with longitudinal stiffness. In contrast, the diaphragm muscle from the mdm mouse showed no difference in the number of mechanomiRs between longitudinal stretch and transverse stretch. This suggests that the diaphragm muscle from the mdm mouse senses greater stiffness in response to longitudinal or transverse directional stretch than the diaphragm from an age-matched WT mouse, as shown previously. These data suggest that the specificity in the mechanosensing ability of the mdm mouse diaphragm might be modified due to its dystrophic nature, perhaps due to disrupted titin cytoskeletal protein [61].

Earlier works conducted in our lab revealed distinct expression patterns of the highly expressed let-7 family mechanomiRs, let-7e-5p and miR-98–5p, as well as their respective target genes related to the extracellular matrix and TGF-β pathways, when comparing WT and mdm mice. Functional studies involving both gain- and loss-of-function of let-7e-5p in myocytes isolated from the diaphragms of control and mdm mice identified Col1a1, Col1a2, Col3a1, Col24a1, Col27a1, Itga1, Itga4, Scd1, and Thbs1 as direct targets of let-7e-5p. Additionally, our results demonstrate that miR-98 exerts a negative regulatory effect on myoblast differentiation. These findings highlight additional molecular contributors to the regulation of diaphragm muscle architecture and myogenesis, offering potential insights into the underlying mechanisms of unexplained muscular dystrophy disorders.

### 4.4. Mechanoregulation of SIRT1 Gene Expression in Diaphragm Muscle

Previous studies have shown a role for sirtuin 1 (*SIRT1*) in the protection against oxidative stress by the forkhead box O (FOXO)-driven superoxide dismutase 2 (Sod2) transcription mechanism [32,62,63]. We have shown for the first time that *SIRT1* is a mechanosensing gene, whose transcriptional activation was regulated by stretch through an early growth response protein 1 (EGR1) in the diaphragm muscle [64]. We have also demonstrated that the resulting transient increase in *SIRT1* expression generates an antioxidative response that contributes to reactive oxygen species scavenging [64]. Our data provided experimental evidence that induction of *SIRT1* by mechanical stretch of the diaphragmatic muscle triggers an antioxidative response through the early response factor EGR1 [64]. It is still an open question if the stretch-dependent induction of *SIRT1* driven by EGR1 is responsible for changes in *SIRT1* gene expression in response to the in vivo biaxial mechanical loading of the diaphragmatic muscle. It would be interesting to explore whether the induction of *SIRT1* would play a role in the antioxidative response that follows exercise training.

### 4.5. Mechanical Dysfunction of the Diaphragm Muscle and Its Clinical Implications

Mechanical ventilation-induced diaphragm dysfunction is an important clinical problem because diaphragmatic muscle weakness is a potential risk factor for the failure to wean patients from mechanical ventilation. The pathogenesis of ventilation-induced diaphragmatic dysfunction has been explored by other investigators [65,66]. In these studies, the mechanically ventilated animals generated excessive production of reactive oxygen species within diaphragm muscle fibers, leading to a cascade of redox-regulated signaling events resulting in decreased protein synthesis. This eventually leads to rapid development of diaphragmatic muscle atrophy and contractile dysfunction [65,66]. The mechanosensing signaling pathways investigated in the above scenario are likely disrupted during a period of prolonged mechanical ventilation. It is important to note, however, that findings from published work in this area cannot prove a causal relationship with complete certainty that disruption of any of the discussed mechanosensing signaling pathways could potentially lead to loss of diaphragm muscle mass in the mechanically ventilated patients or those who suffer from muscular dystrophy. Identifying the specific directional-induced signaling pathways and related genes affected in mechanical ventilator-induced diaphragm atrophy is crucial. Such insights will facilitate the development of future therapeutic interventions targeting diaphragm dysfunction, particularly in individuals undergoing prolonged mechanical ventilation or in elderly populations.

## 5. Concluding Remarks

The diaphragm muscle translates mechanical stimuli into biochemical signals upon stretching during each breathing cycle. Unlike other skeletal muscles, the diaphragm’s multidirectional fiber orientation enables it to stretch both longitudinally and transversely with every breath. Our findings established that stretching the diaphragm in different directions activates distinct mechanotransduction pathways, called anisotropic regulation of mechanosignal transduction pathways. For instance, we observed that longitudinal stretching activates the NF-κB transcription factor, whereas transverse stretching specifically triggers AP-1 transcription factor activation. Both pathways lead to the expression of the *Ankrd2* gene. Additionally, we identified direction-dependent transcription of mechanosensitive microRNAs in response to these stretches. Our published work highlighted the mechanical role of cytoskeleton proteins in modulating the mechanical properties of the diaphragmatic muscle [13,17,23,33,45]. Disruption of this cytoskeletal arrangement results in abnormal gene expression, contributing to a dystrophic muscle phenotype. Nevertheless, the underlying mechanisms connecting disturbed cytoskeletal rearrangement to altered gene expression remain unclear, perhaps due to altered signaling pathways. Elucidating these mechanisms could potentially contribute to developing targeted therapeutic interventions for diaphragm dysfunction associated with various disease and disorder conditions. While most of our observations were obtained from animal models, these findings may potentially apply to humans, given that the diaphragm is a structurally and functionally unique organ found exclusively in mammals.
